# Clinical impact of [^18^F]flutemetamol PET among memory clinic patients with an unclear diagnosis

**DOI:** 10.1007/s00259-019-04297-5

**Published:** 2019-03-26

**Authors:** Antoine Leuzy, Irina Savitcheva, Konstantinos Chiotis, Johan Lilja, Pia Andersen, Nenad Bogdanovic, Vesna Jelic, Agneta Nordberg

**Affiliations:** 1 Department of Neurobiology, Care Sciences and Society, Division of Clinical Geriatrics Center for Alzheimer Research, Karolinska Institutet, Neo, 7th floor, 141 83 Huddinge, Sweden; 2 Medical Radiation Physics and Nuclear Medicine, Karolinska University Hospital, Stockholm, Sweden; 3 Department of Surgical Sciences, Radiology, Nuclear Medicine and PET, Uppsala University, Uppsala, Sweden; 4 Hermes Medical Solutions, Stockholm, Sweden; 5 Clinic for Cognitive Disorders, Theme Aging, Karolinska University Hospital, Stockholm, Sweden

**Keywords:** [^18^F]Flutemetamol, Amyloid PET, Alzheimer’s disease, Diagnostic change, Cholinesterase inhibitors

## Abstract

**Purpose:**

To investigate the impact of amyloid PET with [^18^F]flutemetamol on diagnosis and treatment management in a cohort of patients attending a tertiary memory clinic in whom, despite extensive cognitive assessment including neuropsychological testing, structural imaging, CSF biomarker analysis and in some cases [^18^F]FDG PET, the diagnosis remained unclear.

**Methods:**

The study population consisted of 207 patients with a clinical diagnosis prior to [^18^F]flutemetamol PET including mild cognitive impairment (MCI; *n* = 131), Alzheimer’s disease (AD; *n* = 41), non-AD (*n* = 10), dementia not otherwise specified (dementia NOS; *n* = 20) and subjective cognitive decline (SCD; *n* = 5).

**Results:**

Amyloid positivity was found in 53% of MCI, 68% of AD, 20% of non-AD, 20% of dementia NOS, and 60% of SCD patients. [^18^F]Flutemetamol PET led, overall, to a change in diagnosis in 92 of the 207 patients (44%). A high percentage of patients with a change in diagnosis was observed in the MCI group (*n* = 67, 51%) and in the dementia NOS group (*n* = 11; 55%), followed by the non-AD and AD (30% and 20%, respectively). A significant increase in cholinesterase inhibitor treatment was observed after [^18^F]flutemetamol PET (+218%, 34 patients before and 108 patients after).

**Conclusion:**

The present study lends support to the clinical value of amyloid PET in patients with an uncertain diagnosis in the tertiary memory clinic setting.

## Introduction

Reliable biomarkers are a prerequisite for the early and accurate diagnosis of Alzheimer’s disease (AD) and other dementia disorders. Positron emission tomography (PET) imaging using the metabolic tracer 2-deoxy-2-[^18^F]fluoro-d-glucose ([^18^F]FDG) is a well-established method for the evaluation of functional changes in the brain of patients with dementia disorders, and has been shown to be useful in discriminating the typical pattern of hypometabolism seen in AD from those seen in other dementia disorders [1]. [^18^F]FDG PET shows low accuracy, however, in identifying patients with mild cognitive impairment (MCI) who will convert to different forms of dementia [1, 2]. According to the recent International Working Group 2 criteria [3], [^18^F]FDG PET should be considered a marker of disease progression, in contrast to amyloid-β (amyloid) PET, which is considered a pathophysiological marker.

Since the publication of the first in vivo PET study showing selective imaging of amyloid plaques in AD [4], amyloid PET has proven to be instrumental as a research tool [5]. Beyond this application, amyloid PET holds great potential as a diagnostic aid because of its ability to directly detect a core neuropathological feature of AD, a disease diagnosed with only moderate accuracy, even by expert clinicians [6].

On the basis of convincing findings from phase I–III studies, several fluorine-18 amyloid tracers, including [^18^F]florbetapir (Amyvid®), [^18^F]florbetaben (Neuraceq®) and [^18^F]flutemetamol (Vizamyl®), were commercially developed and approved for use in the exclusion of AD as an underlying cause of cognitive impairment. Although interest in their incorporation into daily practice has been shown to be high among dementia specialists [7] relatively few of the studies addressing their clinical impact [8, 9] have been prospective in nature or performed in the tertiary setting [10–15], with only a subset including patients with MCI in sizeable numbers [11, 15–17]. Moreover, few studies have included CSF biomarkers [10, 13, 18]. The objective of the present study was thus to investigate the impact of [^18^F]flutemetamol PET imaging, in terms of changes in diagnosis and treatment, in patients whose diagnosis remained unclear following clinical assessment at a tertiary memory clinic.

## Materials and methods

### Participants

The study population consisted of 207 patients attending the Clinic for Cognitive Disorders, Theme Aging, Karolinska University Hospital, Stockholm, Sweden. Patients were seen between 2014 and 2018 and had been mainly referred by primary care physicians (GPs), but also from different hospital clinics, owing to different forms of cognitive problems. Some patients were referred from other memory clinics in Sweden to seek a second opinion. Many patients were relatively young (mean age <65 years). Most patients referred by GPs had undergone cognitive testing (e.g. Mini-Mental State Examination, MMSE), structural imaging (CT, or in a few patients, MRI) and blood analysis, while patients referred from other clinics had often undergone less thorough assessments.

At their first visit, patients underwent physical, neurological, psychiatric and cognitive assessments, and a detailed medical history was recorded. Most patients were accompanied by a close relative or friend. Patients were referred for neuropsychological testing, CT/MR imaging and CSF sampling, and some underwent apolipoprotein E (APOE) genotyping, electroencephalography, speech/language testing and [^18^F]FDG PET. Diagnoses were based on a consensus meeting between specialists in cognitive disorders, clinical neuropsychologists and specialist nurses. Diagnostic categories included MCI [19, 20], AD [21, 22], dementia of unclear aetiology (not otherwise specified, dementia NOS) [22], dementia due to a non-AD disorder, including dementia with Lewy bodies (DLB) [23], frontotemporal dementia (FTD) [24] and vascular dementia [25], and subjective cognitive decline (SCD) [26].

Patients were referred for amyloid imaging with [^18^F]flutemetamol PET because of an uncertain diagnosis despite extensive cognitive and biomarker-based assessments, and [^18^F]flutemetamol PET was generally performed either directly after initial diagnosis or later, during clinical follow-up. Following the [^18^F]flutemetamol PET scan, patients revisited the Clinic for Cognitive Disorders and were informed of their results, any possible changes in diagnosis and the management plan by a specialist in dementia disorders. The diagnostic categories before and after [^18^F]flutemetamol PET were the same, except for the addition of prodromal AD in a subset of patients with MCI [3].

### Neuropsychological assessments

Most patients completed a large battery of neuropsychological tests covering different cognitive domains [27]. These included the MMSE as well as components of the Wechsler Adult Intelligence Scale, Revised (WAIS-R; information and similarities, logical memory, block design and digit symbol), figure classification, subtest of the Synonyms Reasoning Block Test (SRB2), Rey Auditory Verbal Learning Test (RAVLT), copying and memory subtests of the Rey-Osterrieth Complex Figure Test (ROCFT), parts A and B of the Trail Making Test (TMT), and/or the Verbal Fluency Test (FAS).

### Structural imaging

Structural CT or T1-weighted MR imaging was performed at various radiology departments in Stockholm using different platforms and protocols. Cerebral atrophy and white matter lesions were assessed clinically by experienced neuroradiologists at the Department of Radiology, Karolinska University Hospital, according to standard visual rating scales. Atrophy of the medial temporal lobe was evaluated using the medial temporal atrophy (MTA) scale [28]. Overall cortical atrophy was assessed using the global cortical atrophy (GCA) scale [29]. White matter hyperintensities were scored using the Fazekas scale [30].

### CSF biomarkers

CSF samples were collected via lumbar puncture from 152 out of the 207 patients (73%) under nonfasting conditions as part of routine memory assessment. The lack of CSF biomarkers in 55 patients was due to the use of anticoagulants in 18 patients, spinal stenosis or related problems in 6, refusal to undergo CSF sampling in 12, and technical problems in 19. The CSF samples were routinely analysed at the Clinical Neurochemistry Laboratory, Sahlgrenska University Hospital, Mölndal, Sweden, for Aβ_1-42_, t-tau and p-tau, using commercially available ELISAs (INNOTEST; Fujirebio, Ghent, Belgium). Internal cut-off values of 550 ng/L for Aβ_1-42_, 400 ng/L for t-tau and 80 ng/L for p-tau were used.

### PET investigations

[^18^F]FDG and [^18^F]flutemetamol PET investigations were performed at the Department of Medical Radiation Physics and Nuclear Medicine Imaging, Karolinska University Hospital, Stockholm, Sweden, using a Biograph mCT PET/CT scanner (Siemens/CTI, Knoxville, TN).

### [^18^F]FDG PET

[^18^F]FDG PET was performed as part of the clinical assessment in 78 out of the 207 patients (38%). These studies were performed prior to [^18^F]flutemetamol PET (median 5 months, interquartile range, IQR, 3–12 months) in 68 patients (87%) and after [^18^F]flutemetamol PET (median 2 months, IQR 0.5–3 months) in 9 patients. [^18^F]FDG PET was performed as a 10-min or 15-min list-mode scan 30 to 45 min after injection of 2–3 MBq/kg. All appropriate corrections, including point spread function, scatter and time-of-flight were applied, with a low-dose CT scan used for attenuation correction. Images were reconstructed using ordered subsets expectation maximization (OSEM; five iterations, 21 subsets, 2.0 mm gaussian filter), yielding an effective spatial resolution of 3.0 mm.

According to clinical routine assessment, summation images were visually analysed, supported by a semiquantitative analysis in the form of standardized uptake value ratios (SUVR) based on automatically generated regions of interest (ROI; cortical and subcortical regions, normalized to whole brain) and voxelwise Z-score stereotactic surface projection images (Siemens *syngo*.via software). Metabolic patterns were classified as typical of AD, as suggestive but not typical of AD, as consistent with other non-AD neurodegenerative diseases (e.g. FTD or DLB) or as nonspecific in the case of minor patchy focal changes.

### [^18^F]Flutemetamol PET

[^18^F]Flutemetamol PET was performed as a 20-min list-mode scan 90 min after injection of 185 MBq [^18^F]flutemetamol. Data were corrected and images reconstructed in an identical way to the [^18^F]FDG PET studies. [^18^F]Flutemetamol summation images were visually assessed as positive (abnormal) or negative (normal) by a board-certified nuclear medicine physician with later reassessment by either a neuroradiologist or a physician with experience in neuroimaging who had successfully completed an electronic training programme developed by GE Healthcare for the interpretation of [^18^F]flutemetamol images [31]. In cases of disagreement between raters, a consensus was achieved between readers during the re-read session.

In addition to visual reads, semiquantitative analysis of [^18^F]flutemetamol uptake was performed using an automated ROI-based approach (Hermes Medical Solutions) [32], with SUVR calculated using an isocortical composite ROI, comprising brain regions typically associated with high amyloid load in AD (frontal, lateral temporal, cingulate and parietal cortices), using the pons as the reference region. Amyloid-positivity was defined using an a priori SUVR cut-off value of 0.60, based on separation from cognitively normal controls [33].

### Statistical analysis

Characteristics were compared between diagnostic groups using Kruskal Wallis analysis of variance (ANOVA) and the Wilcoxon signed ranks test for continuous variables. Categorical variables were assessed using Fisher’s exact test. Agreement between visual and SUVR-based [^18^F]flutemetamol classifications, as well as interrater agreement for visual readings, were assessed using percentage agreement and Cohen’s kappa. The proportions of patients showing changes in diagnosis and drug treatment were assessed using a one-sample proportions test. All statistical tests were performed using R v. 3.3.2 (The R Foundation for Statistical Computing), with two-sided *p* values <0.05 considered to indicate significance.

## Results

**Fig. 1:**
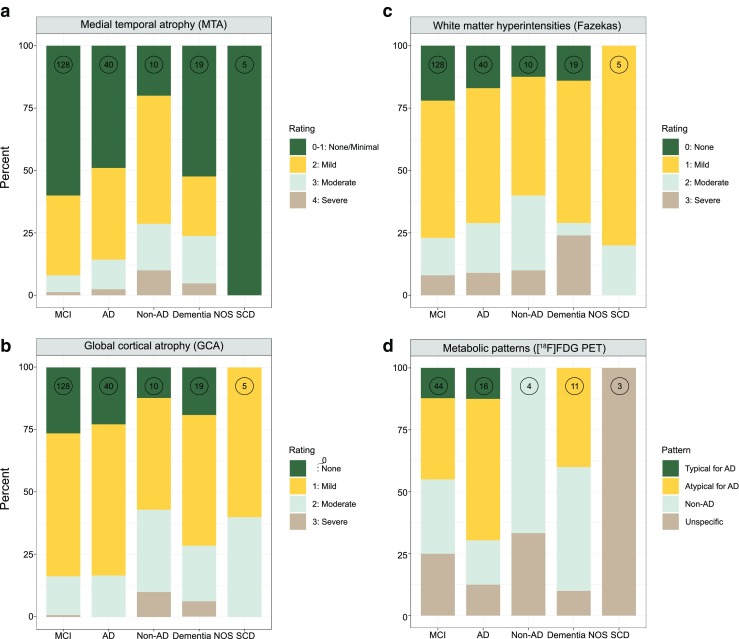
CT/MRI-based ratings of atrophy and white matter changes and [^18^F]FDG PET metabolic patterns shown as the distributions of medial temporal atrophy (**a**), global atrophy (**b**), white matter changes (**c**) and metabolic patterns (**d**) based on the diagnoses made prior to [^18^F]flutemetamol PET (the number at the top of each column indicates the number of patients)

The demographic, clinical and biomarker findings in the 207 patients in whom [^18^F]flutemetamol PET was performed due to diagnostic uncertainty are presented in Table 1. Most patients received an initial diagnosis of MCI (131, 63%), followed by AD (41, 20%), dementia NOS (20, 10%), non-AD (10, 5%) and SCD (5, 2%). Figure 1 shows the structural imaging-based ratings for atrophy and white matter changes in the different patient groups before amyloid PET. MTA scores of 0 or 1, indicating no or minimal atrophy, respectively, were predominant across the MCI, AD, dementia NOS and SCD groups. In contrast, MTA scores indicating mild atrophy were noted in half of the patients with non-AD disorders. In terms of GCA and white matter lesions, most patients showed mild changes. [^18^F]FDG PET imaging was performed in 78 of the 207 patients (38%; Table 1, Fig. 1d). A metabolic pattern suggestive but not typical of AD was the most common finding in those with a diagnosis of MCI or AD prior to [^18^F]flutemetamol PET (16 patients, 36%, and 9 patients, 56%, respectively); in the remaining groups, patterns not typical of AD or suggestive of a non-AD disorder were predominant (non-AD, 4 patients, 100%; dementia NOS, 10 patients, 91%), with only minor patchy changes seen in patients with SCD. Similar findings were obtained using the diagnoses obtained after [^18^F]flutemetamol PET, with only 11 AD patients (14%) showing a typical metabolic pattern.

**Table 1:** Demographic, clinical and biomarker data using diagnoses obtained prior to [^18^F]flutemetamol PET

		MCI	AD	Non-AD disorder	Dementia NOS	SCD
Number of patients		131	41	10	20	5
Age (years), mean (SD)		64.5 (8.6)	65.2 (8.7)	67.6 (5.2)	62.3 (9.5)	68.4 (8.3)
Sex, female/male (*n*/*n*)		76/55	27/14	5/5	10/10	3/2
MMSE^a^		25.6 (3.7)	24.5 (3.7)	23.4 (3.8)	22 (5.5)	29 (1.2)
*APOE* ε4						
Number of patients with data available		75	27	4	9	5
Number (%) positive		42 (56)	15 (56)	2 (50)	2 (22)	2 (40)
CSF biomarkers						
Number of patients with data available		86	38	9	15	4
Aβ_1-42_ (ng/L), mean (SD)		595 (164)	612 (325)	627 (198)	678 (272)	685 (98.4)
p-tau (ng/L), mean (SD)^b^		52 (29)	59 (23)	49 (23)	39.9 (24.5)	73 (19.8)
t-tau (ng/L), mean (SD)^c^		369 (228)	429 (201)	506 (152)	299 (289)	549 (241)
CT/MRI scales						
Number of patients with data available		128	40	10	19	5
MTA (*n*)^d^	0/1^k^	76	20	2	10	5
	2	39	14	5	6	0
	3	11	5	2	2	0
	4	2	1	1	1	0
GCA (*n*)^e^	0	32	8	1	4	0
	1	75	25	5	9	3
	2	20	7	3	5	2
	3	1	0	1	1	0
Fazekas scale (*n*)^f^	0	28	7	1	3	0
	1	68	21	6	10	4
	2	21	8	2	1	1
	3	11	4	1	5	0
[^18^F]FDG metabolic patterns^g^						
Number of patients with data available		44	16	4	11	3
Typical of AD (*n*)		4	2	0	0	0
Not typical of AD (*n*)		16	9	0	5	0
Non-AD (*n*)		13	3	2	5	0
Nonspecific (*n*)		11	2	2	1	3
[^18^F]Flutemetamol						
Number (%) positive on visual assessment^h^		69 (53)	28 (68)	2 (20)	4 (20)	3 (60)
Global SUVR, mean (SD)^I^		0.61 (0.19)	0.71 (0.18)	0.47 (0.05)	0.52 (0.18)	0.64 (0.18)
Number (%) positive by SUVR^j^		67 (51)	28 (68)	2 (20)	4 (20)	3 (60)

*AD* Alzheimer’s disease, *GCA* global cortical atrophy, *MCI* mild cognitive impairment, *MMSE* Mini-Mental State Examination, *MTA* medial temporal atrophy, *NOS* not otherwise specified, *SCD* subjective cognitive decline, *SUVR* standardized uptake value ratio

^a^MMSE: dementia NOS < AD (*p* < 0.01); MCI, AD, non-AD, dementia NOS < SCI (*p* < 0.01)

^b^ p-tau AD > MCI and dementia NOS (*p* < 0.05); SCI > dementia NOS (*p* < 0.05)

^c^t-tau: AD > dementia NOS (*p* < 0.01)

^d^MTA (*0/1* none/minimal, *2* mild, *3* moderate, *4* severe): MCI vs. non-AD (*p* < 0.01); AD vs. non-AD (*p* < 0.01); non-AD vs. dementia NOS (*p* < 0.05)

^e^GCA: *0* none, *1* mild, *2* moderate, *3* severe

^f^Fazekas scale (white matter hyperintensities): *0* none, *1* mild, *2* moderate, *3* severe

^g^[^18^F]FDG metabolic patterns: AD vs. SCI (*p* < 0.05), dementia NOS vs. SCI (*p* < 0.01)

^h^Visual assessment: MCI > dementia NOS (*p* < 0.01), AD > non-AD (*p* < 0.01), AD > dementia NOS (*p* < 0.001)

^I^SUVR: MCI > dementia NOS (*p* < 0.05), AD > MCI (*p* < 0.01), non-AD (*p* < 0.01), dementia NOS (*p* < 0.001)

^j^SUVR positivity (SUVR defined using a global cortical cut-off value of >0.60): MCI > dementia NOS (*p* < 0.01), AD > non-AD (*p* < 0.01) AD > dementia NOS (*p* < 0.001)

^k^MTA 0 and 1 are considered normal; these categories were therefore combined

**Fig. 2:**
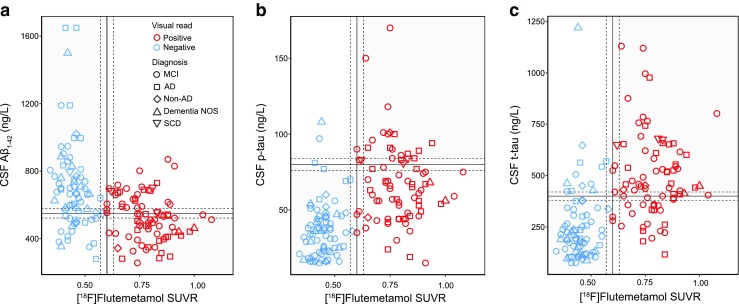
Relationships between CSF biomarkers and isocortical composite [^18^F]flutemetamol SUVR. The *vertical lines* mark the cut-off value of 0.60 for isocortical composite [^18^F]flutemetamol SUVR; the *horizontal lines*mark the cut-off values for Aβ_1-42_ (**a** <550 pg/mL), p-tau  (**b** >80 pg/mL) and t-tau (**c** >400 pg/mL); the *dashed lines* indicate borderline zones (within 5% of the cut-off values); and the *grey and white quadrants* indicate concordance and discordance between biomarkers, respectively

CSF was sampled in 152 patients according to the Swedish clinical practice guidelines for memory assessment at specialist memory clinics. Overall, 103 patients (68%) showed abnormal CSF biomarkers: 24% showed only abnormal Aβ_1-42_, 21% showed abnormal Aβ_1-42_ in combination with elevated t-tau or p-tau, and 32% showed negative CSF; a further 23% showed only abnormal tau. The main reasons for performing amyloid PET were a clinical suspicion of AD in combination with either a negative or an unclear (i.e. isolated positive or borderline Aβ_1-42_ or tau) CSF profile (117 patients, 57%) or the absence of CSF samples (55 patients, 27%). A clinically unclear picture of memory decline in combination with a CSF profile indicating AD (low Aβ_1-42_ and one or both tau markers positive) was the third most common reason (32 patients, 15%). Table 2 and Fig. 2 show the relatively poor agreement between CSF biomarkers and [^18^F]flutemetamol PET (Aβ_1-42_, 66%; p-tau, 76%; t-tau, 77%).

**Table 2:** Agreement between CSF positivity and [^18^F]flutemetamol PET positivity using dichotomized measures based on the diagnoses made before [^18^F]flutemetamol PET

CSF	[^18^F]Flutemetamol PET^a^	CSF biomarker^b^	Number (%) of patients positive				
			MCI (*n* = 86^c^)	AD (*n* = 38^c^)	Non-AD disorder (*n* = 9^c^)	Dementia NOS (*n* = 15^c^)	SCD (*n* = 4^c^)
Positive	Positive	Aβ_1-42_	25 (29)	19 (50)	2 (22)	2 (13)	–
		p-tau	30 (35)	19 (50)	2 (22)	2 (13)	3 (75)
		t-tau	29 (34)	20 (52)	3 (33)	2 (13)	3 (75)
	Negative	Aβ_1-42_	11 (13)	3 (8)	3 (33)	2 (13)	–
		p-tau	3 (3)	4 (10)	2 (22)	3 (20)	–
		t-tau	3 (3)	3 (8)	1 (11)	3 (20)	–
Negative	Positive	Aβ_1-42_	23 (27)	6 (16)	–	–	3 (75)
		p-tau	18 (21)	6 (16)	–	–	–
		t-tau	19 (22)	6 (16)	–	–	–
	Negative	Aβ_1-42_	27 (31)	10 (26)	4 (45)	11 (74)	1 (25)
		p-tau	35 (41)	9 (24)	5 (56)	10 (67)	1 (25)
		t-tau	35 (41)	9 (24)	5 (56)	10 (67)	1 (25)

Concordance (both biomarkers positive or negative): CSF-positive/PET-positive and CSF-negative/PET-negative. Discordance (only one of two biomarkers positive): CSF-positive/PET-negative (isolated CSF positivity) and CSF-negative/PET-positive (isolated PET positivity)

^a^The cut-off value used for [^18^F]flutemetamol SUVR was 0.60, in combination with visual assessment

^b^The cut-off values used to binarize CSF biomarkers were <550 ng/L for Aβ_1-42_, >80 ng/L for p-tau and >400 ng/L for t-tau

^c^Number of patients in whom CSF was sampled

There was high agreement between raters in the visual analysis of the [^18^F]flutemetamol PET scans (198/207, 96%; Cohen’s kappa = 0.92). In 37 visual readings, however, one of the two readers had some difficulty in defining the [^18^F]flutemetamol PET scan as positive or negative; this finding was similarly distributed between experienced and less experienced readers (20 and 17 readings, respectively). In four scans, both readers were uncertain. Overall, some uncertainties were observed in 11% of readings. Agreement between visual and semiquantitative assessment approaches was achieved in all cases. Using both visual and SUVR-based classifications, a higher proportion of patients were rated as [^18^F]flutemetamol-positive in the preamyloid PET MCI and AD patients than in the dementia NOS patients (*p* < 0.01), and in the AD patients as compared with the non-AD patients (*p* < 0.05).

**Fig. 3:**
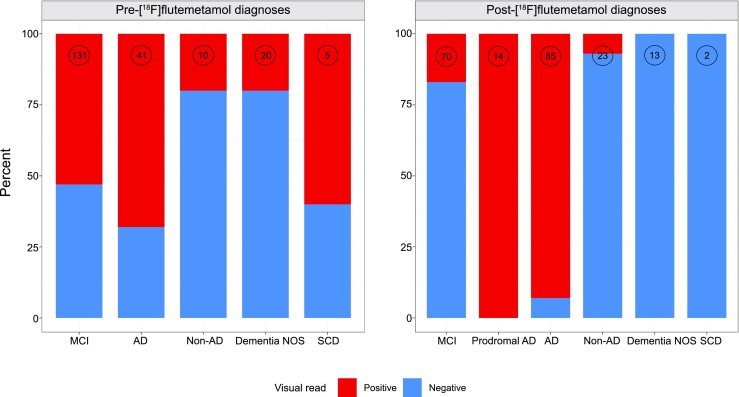
Visual [^18^F]flutemetamol ratings in the various diagnostic groups before (**a**) and after (**b**) [^18^F]flutemetamol PET (the number at the top of each column indicates the number of patients). *Red* [^18^F]flutemetamol-positive, *blue* [^18^F]flutemetamol-negative

On visual evaluation of the [^18^F]flutemetamol PET scans, amyloid positivity was found in 69 of 131 patients (53%) with an initial diagnosis of MCI, in 28 of 41 (68%) with AD, in 2 of 10 (20%) with a non-AD disorder, in 4 of 20 (20%) with dementia NOS and in 3 of 5 (60%) with SCD (Table 1). Amyloid status in the diagnostic groups before and after [^18^F]flutemetamol PET as well as changes in diagnosis are summarized in Table 3 and Fig. 3, respectively. As shown in Fig. 3, the majority of patients with a diagnosis of MCI after [^18^F]flutemetamol were amyloid-negative. The vast majority of patients with an initial diagnosis of MCI who were amyloid-positive received a diagnosis of prodromal AD or AD (54 of 60, 90%). In patients with an initial diagnosis of AD, the diagnosis was dismissed in seven amyloid-negative patients**. **Finally, all patients with an initial diagnosis of dementia NOS group, and almost all patients with a non-AD disorder were amyloid-negative. Overall, [^18^F]flutemetamol PET led to a significant change in diagnosis (92 patients, 44%; *p* < 0.05). Among the patients with MCI, dementia NOS, AD and a non-AD disorder, the highest percentage change in diagnosis was observed in those with MCI (67 patients, 51%) as well as in those with dementia NOS (11 patients, 55%), while a smaller percentage change was seen in those with a non-AD disorder and those with AD (3 patients, 30%, and 8 patients, 20%, respectively). Among the five patients with SCD, in three with a positive [^18^F]flutemetamol scan the initial diagnosis was revised to MCI, prodromal AD and AD, respectively, on clinical follow-up.

**Table 3:** Change in diagnosis following [^18^F]flutemetamol PET

Initial diagnosis	Change	[^18^F]Flutemetamol PET^a^	Number (%) of those with change	Diagnosis after [^18^F]flutemetamol PET
MCI	67/131 (51%)	Positive	58 (87%)	1 non-AD (DLB), 13 prodromal AD, 44 AD
		Negative	9(13%)	1 AD, 3 dementia NOS, 5 non-AD (4 VaD, 1 PSP)
AD	8/41 (20%)	Positive	1 (12.5%)	Non-AD (DLB)
		Negative	7 (87.5%)	1 dementia NOS, 2 non-AD (DLB, FTD), 4 MCI
Non-AD	3/10 (30%)^b^	Positive	2 (67%)	AD
		Negative	1 (33%)	MCI
Dementia NOS	11/20 (55%)	Positive	4 (36%)	AD
		Negative	7 (64%)	Non-AD (1 DLB, 3 VaD, 3 FTD)
SCD	3/5 (60%)	Positive	3 (100%)	MCI, prodromal AD, AD
		Negative	–	–

*DLB* dementia with Lewy bodies, *FTD* frontotemporal dementia, *PSP* progressive supranuclear palsy, *VaD* vascular dementia

^a^[^18^F]Flutemetamol PET status (positive/negative) was based on visual assessment supported by SUVR findings

^b^In two non-AD patients with DLB the initial diagnosis was FTD

The outcome of amyloid PET led not only to a change in diagnosis but also to more patients receiving treatment with cholinesterase inhibitors (ChEIs). Nine patients with MCI (seven amyloid-positive), 22 with prodromal AD/AD (16 amyloid-positive) and 3 with non-AD/dementia NOS (one amyloid-positive) were receiving treatment with various ChEIs prior to amyloid PET. After amyloid PET and revision of initial diagnoses, 92 patients with prodromal AD/AD (87 amyloid-positive), 8 with MCI (4 amyloid-positive) and 9 with non-AD/dementia NOS (2 amyloid-positive) received ChEI treatment. ChEI treatment was used in 34 patients prior to amyloid PET and in 109 patients after amyloid PET, an increase of 75 patients (+218%; *p* < 0.001). Treatment was discontinued following [^18^F]flutemetamol PET in one amyloid-negative patient with MCI due to cholinergic side effects. Of the 109 patients receiving ChEI treatment, 93 (85%) were amyloid-positive, and 92 of 99 patients (93%) had a diagnosis of prodromal AD/AD after [^18^F]flutemetamol PET, while only 8 of 72 patients with MCI and 9 of 36 (25%) with non-AD/dementia NOS (including those with DLB) received ChEI treatment.

## Discussion

The rapid development of molecular imaging techniques has enabled the in vivo study of the pathophysiology underlying different neurodegenerative diseases, including AD. Amyloid imaging has reached clinical use in memory clinics. However, most research studies published to date have included selected research populations. The present study included an unselected cohort of 207 patients, in whom extensive neuropsychological and, in various subsets, biomarker-based assessments could not provide a sufficiently certain clinical diagnosis. The main reasons for amyloid PET in our cohort were the clinical suspicion of AD accompanied by unclear or negative CSF findings, the absence of CSF biomarkers (due to contraindications, patient refusal or technical difficulties) and an ambiguous clinical presentation in the context of a CSF profile indicative of AD (low Aβ_1-42_ and elevated tau).

Despite the fact that [^18^F]FDG PET is a well-established diagnostic tool in the work-up of dementia disorder patients, it did not provide an adequate differential diagnosis in the present cohort. We estimate that this cohort of patients, requiring complementary amyloid PET after an extensive clinical work-up including neuropsychological testing, MRI, CSF biomarker analysis and other additional examinations, represents approximately 10% of all new referrals to our academic memory clinic (approximately 500 to 600 per year).

In this study, we assessed the incremental value of amyloid PET in the work-up of patients being followed in a tertiary specialist setting due to cognitive impairment. The largest patient group was MCI patients of whom 69 (53%) were [^18^F]flutemetamol PET-positive. A positive [^18^F]flutemetamol PET scan in this group led mainly to a diagnosis of prodromal AD/AD, while a negative scan, with a few exceptions, led to a diagnosis of non-AD/dementia NOS, or (in a subset of MCI patients) to retention of the original diagnosis. Overall, amyloid PET led to a significant change in diagnosis in 44% of patients and an increase in the use of AD drug treatment, from 34 to 109 of 207 patients (16% to 53%). On the basis of our study design, in which amyloid PET results were used to revise initial diagnoses and treatment plans, our findings provide an estimate of the incremental value of this type of investigation in the clinical setting. Thus, this study fulfils the requirements of a phase 4 study as defined by the five-phase biomarker validation framework recently introduced to the field [34, 35]. This type of study is vital to assess the utility of amyloid PET and to accelerate the development of evidence-based guidelines for its clinical use [36].

In the present study, the concordance rates observed between [^18^F]flutemetamol PET and CSF Aβ_1-42_ were lower than those reported to date from the ADNI (Alzheimer’s Disease Neuroimaging Initiative) and the Swedish BioFINDER (Biomarkers for Identifying Neurodegenerative Disorders Early and Reliably) studies [37–41], in which the composition of the cohorts largely resembles that found in clinical trials. The difference in observations is due to the fact that in the present study only patients with an unclear diagnosis following extensive assessment were recruited for amyloid imaging. Although our study, by design, was predisposed to discordance, it draws attention to potential complications in implementing the recent AD biomarker classification scheme [42]: based on categorical and biomarker analyses, this framework assumes that measures of amyloid based on CSF analysis and PET are interchangeable. Although disagreement between imaging and CSF analysis may be a reflection of how cut-off values are established—with European laboratories generally using Aβ_1-42_ cut-off values that are too low [18]—the use of a more lenient cut-off value (647 ng/L) derived from a recent study using the same CSF INNOTEST assay [40] resulted in the same overall level of concordance; though an increase in concordant-positive subjects was seen, this was accompanied by an increase in the number of subjects showing isolated CSF positivity. There is evidence, however, to suggest that higher cut-off values for Aβ_1-42_ may prove appropriate. Further studies addressing this, including comparison with Aβ_1-42_/tau ratios, are warranted*. *While many subjects may show concordance in the long term [42], more work is required to integrate and compare CSF analysis and amyloid PET so as to develop more refined guidelines for how these biomarkers are to be used and interpreted in the diagnostic work-up of patients with dementia disorders [43], including considerations related to differences in amyloid processing and neurodegeneration seen across atypical forms of AD [44] and the value of Aβ_1-42_ ratios with shorter isoforms [45, 46] and tau/Aβ_1-42_ [47].

The percentage of patients with a change in diagnosis observed in this study (44%) was higher than the average of those found in previous studies [48], although somewhat lower than in other studies [10, 11]. This most likely reflects differences in the composition of the study cohorts; in the present study, diagnostic uncertainty remained despite extensive neuropsychological testing, imaging and biomarker investigations. Our study cohort therefore most likely included a higher proportion of patients with atypical disease, an assertion supported by the number of MCI and dementia NOS patients with an unchanged diagnosis. We consider, however, that the most important finding was the high percentage of amyloid-positive MCI patients who received a change in diagnosis to prodromal AD or AD, and thus began treatment with ChEIs. Although currently not included in the appropriate use criteria for amyloid PET [49], patients with SCD are increasingly discussed as an at-risk population given the association between SCD and cognitive decline in the context of biomarker evidence for AD [50, 51]. Indeed, our findings that most such patients showed abnormal CSF findings and clinical progression at follow-up suggest that this group is important in the context of best practice guidelines.

In our cohort, amyloid PET led to significant changes in the management of patients, including treatment with ChEIs, with a more than threefold increase in the number of patients receiving ChEI treatment. This increase was primarily accounted for by amyloid-positive patients with a diagnosis after [^18^F]flutemetamol PET of prodromal AD/AD. Similarly, amyloid-positive patients with a diagnosis after [^18^F]flutemetamol PET of AD comprised the majority of those receiving ChEIs prior to undergoing amyloid PET. The small number of [^18^F]flutemetamol-positive MCI and non-AD (DLB) patients receiving cholinergic drugs probably reflects varying adherence by physicians to the diagnostic codes (i.e. MCI, amyloid-positive, versus prodromal AD, a research term not currently recognized in dementia care guidelines) and evidence suggesting that this drug class is of benefit in DLB [52]. Among the few amyloid-negative patients, treatment, whether initiated prior to or after [^18^F]flutemetamol PET, was related to CSF Aβ_1-42_ positivity (seen in close to half of such patients) and/or physician-specific treatment beliefs.

The present study underlines the value of amyloid PET when CSF biomarkers are not consistent or when CSF sampling is not possible due to the use of anti-coagulants, other medical reasons, or patient refusal of lumbar puncture. In this population of patients it has also been demonstrated that [^18^F]FDG PET is not always useful, especially in those with MCI. A negative amyloid PET study is important information for an MCI patient and often results in a decease in the use of resources due to fewer ancillary investigations. In patients with aetiologically unclear dementia or non-AD, amyloid PET could provide important diagnostic information, as was illustrated in the present study with a 55% change in diagnosis among dementia NOS patients. Although the fact that the generally rather extensive investigations undergone by the patients in this study help to establish the clinical usefulness of [^18^F]flutemetamol PET, it must also be underlined that our findings may not be generalizable to older patients or those attending less specialized clinical centres. Although incorporating cost-effectiveness parameters associated with amyloid imaging was beyond the scope of the present investigation, further studies addressing this, including the optimal stage (early versus late) for the use of amyloid PET in the diagnostic work-up of patients, are crucial.

### Conclusion

In summary, our findings indicate that amyloid PET imaging with [^18^F]flutemetamol had a significant impact in terms of change of diagnosis, management and drug treatment when added to the work-up of patients with an uncertain diagnosis followed in the setting of a tertiary memory clinical. These findings highlight the clinical value of amyloid PET in patients with cognitive impairment of unclear aetiology.
